# Quality, Nutritional Properties, and Glycemic Index of Colored Whole Wheat Breads

**DOI:** 10.3390/foods12183376

**Published:** 2023-09-08

**Authors:** Hamit Koksel, Buket Cetiner, Vladimir P. Shamanin, Z. Hazal Tekin-Cakmak, Inna V. Pototskaya, Kevser Kahraman, Osman Sagdic, Alexey I. Morgounov

**Affiliations:** 1 Department of Nutrition and Dietetics, Health Sciences Faculty, Istinye University, İstanbul 34010, Türkiye; hamit.koksel@istinye.edu.tr (H.K.); hazal.cakmak@yildiz.edu.tr (Z.H.T.-C.); 2 Department of Agronomy, Breeding and Seed Production of the Agrotechnological Faculty, Omsk State Agrarian University, 1 Institutskaya pl., Omsk 644008, Russia; vp.shamanin@omgau.org (V.P.S.); iv.pototskaya@omgau.org (I.V.P.); 3 Department of Quality and Technology, Field Crops Central Research Institute, Ankara 06170, Türkiye; buket.cetiner@tarimorman.gov.tr; 4 Department of Food Engineering, Faculty of Chemical and Metallurgical Engineering, Davutpasa Campus, Yildiz Technical University, İstanbul 34349, Türkiye; osagdic@yildiz.edu.tr; 5 Department of Material Science and Nanotechnology Engineering, Abdullah Gul University, Kayseri 38080, Türkiye; kevser.kahraman@agu.edu.tr; 6 Science Department, S. Seifullin Kazakh Agrotechnical University, Astana 010011, Kazakhstan

**Keywords:** purple wheat, blue wheat, bread quality, glycemic index, dietary fiber

## Abstract

The main aim of this study was to investigate the quality and nutritional properties (dietary fiber, phenolic, antioxidant contents, and glycemic index) of breads made from whole wheat flours of colored wheats. White (cultivar Agronomicheskaya 5), red (Element 22), purple (EF 22 and Purple 8), and blue (Blue 10) colored wheats were used in the study. The whole wheat flours of Blue 10 and Purple 8 had higher farinograph stability, lower softening degree, and higher quality numbers indicating that they had better rheological properties. Bread produced from whole wheat flour of blue-colored grain had significantly higher loaf volume and better symmetry, crust color, crumb cell structure, and softness values among others (*p* < 0.05). The whole wheat bread produced using Element 22 had the highest crust and crumb L* color values, while Purple 8 and EF 22 had the lowest crust and crumb L* color values, suggesting that purple-colored grains have a tendency to make whole wheat bread with darker crust and crumb color. Bread produced from cultivar Blue 10 had the lowest firmness values while Element 22 had the highest firmness values. The highest total phenolic content and antioxidant capacity values were obtained from the whole wheat bread sample from purple-colored wheat (Purple 8). The whole wheat flour of Element 22 had the highest total dietary fiber content among all samples (*p* < 0.05). The differences between whole wheat bread samples in terms of total dietary fiber and glycemic index were not statistically significant. The results of the present study indicated that colored wheats can be used to produce whole wheat breads with higher nutritional properties and acceptable quality characteristics.

## 1. Introduction

Wheat is one of the most cultivated, traded, and consumed cereals worldwide with an annual cultivation area of 217 million ha [1]. It was domesticated more than 12,000 years ago in the Fertile Crescent and represents an important part of the human diet. Most of the healthy components found in the bran and germ fractions are reduced during the grain-milling process. Whole wheat flour consists of the original composition of bran, germ, and endosperm of wheat grain. Whole wheat products are relatively rich in vitamins, essential minerals, phenolic compounds, dietary fiber, and antioxidants that have been linked to disease prevention [2,3,4,5]. Hence, whole wheat flour offers various nutritional benefits compared to refined wheat flour. Studies also indicate that consuming dietary fiber-containing foods has consistent effects on hunger and satiety. In general, less hunger is experienced after consuming high-fiber meals than after the low-fiber equivalents [6]. 

Due to increasing nutritional awareness and changing consumer demands, there is a growing tendency towards nutritionally balanced and health-promoting diets. Future challenges include meeting the food and nutritional needs of growing populations. The content of phytochemicals is generally limited in white- or red-grained wheat varieties while they occur more consistently in the so-called pigmented/colored varieties [7]. The colored wheat provides nutritional products with higher flavonoids (e.g., anthocyanin), phenolic acids, carotenoids, and other compounds with antioxidant activities that are primarily located in the pericarp and aleurone layers [8,9,10]. Owing to the presence of these compounds and dietary fibers, colored wheats exhibit antioxidant properties that basically help in protecting against degenerative diseases [11]. Pigmented or colored wheats (*Triticum aestivum*) contain various phytochemicals, responsible for various health advantages such as against obesity, type 2 diabetes, cardiovascular disease, and cancer [12]. The purple color is due to the anthocyanins accumulated in the pericarp while the blue color is caused by anthocyanins accumulated in the aleurone layer [11]. The colored wheats provide a novel option of targeting malnutrition by enhancing the antioxidants such as carotenoids, and polyphenols (e.g., phenolic acids, flavonoids, especially anthocyanins). Moreover, colored wheats have opened up a hidden avenue for providing additional value to wheat-based products [10].

Although there are a number of studies investigating various aspects of colored wheats, we have not encountered any study investigating both the grain quality (physical characteristics, protein and gluten contents, farinograph properties) and nutritional properties (dietary fiber, phenolics, antioxidant contents, and glycemic index) of the breads made from whole wheat flours of colored wheats in the same study. The main aim of this study was to investigate the quality (physical characteristics, protein and gluten contents, farinograph properties) and nutritional properties (dietary fiber, phenolics, antioxidant contents, and glycemic index) of whole wheat breads produced by using blue, purple, and white/red colored wheats.

## 2. Materials and Methods

### 2.1. Materials

**Figure 1:**
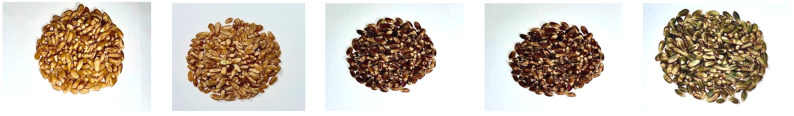
Grain of white-grained variety Agronomicheskaya 5, red-grained variety Element 22, purple-grained variety EF 22 and line Purple 8, blue-grained line Blue 10.

Bread wheat genotypes obtained from Omsk State Agrarian University (Russian) were used in the study. For the first time under conditions of Western Siberia, the variety EF 22 and line Element 22-PurplePF (8) with a purple grain color, as well as the blue-grained wheat line Element_22-Blue4Th (10) (Figure 1), were obtained by crossing variety Element 22 with an anthocyanin biosynthesis gene donor. Variety EF 22 and line Element 22-PurplePF (8) are sister lines that were obtained by individual selections from the hybrid combination Element 22 *2/i:S29PF.

Near-isogenic line, i:S29_Pp-D1Pp3PF (abbreviation—i:S29PF), inherited a dark-red coleoptile color and purple pericarp color from their donor spring breeding line Purple Feed (k-49426, Canada), in the genetic background of red-grained Russian spring bread wheat cv. Saratovskaya 29 (S29) were developed early by Arbuzova et al. [13], and was mapped by Tereshchenko et al. [14]. This line with dominant alleles of genes Pp-1D and Pp3 on chromosomes 7D and 2A was used as a donor of the anthocyanin pericarp pigmentation.

The donor of the anthocyanin pigmentation in the aleurone layer of the grains was a substituted blue-grain wheat line based on the Saratovskaya 29 variety, carrying the *Thinopyrum ponticum* (Podp., syn. *Agropyron elongatum* Host., *Elytrigia pontica* Podp., Holub) chromosome 4Th instead of chromosome 4D (s:S29_4Th/4D), containing the dominant allele of the Ba gene (blue aleuron) [15].

Cultivar Agronomicheskaya 5 has white and Element 22 has red grain colors. The *cv*. Agronomicheskaya 5 with a white grain color was developed at Omsk SAU in the framework of the international KASIB program by using marker-assisted selection and individual selections from the shuttle breeding hybrid population—Sonata *2//TAM 200/Turaco (CIMMYT, Mexico). The varieties and lines used in this study have a spring growth habit, their origins and grain color values are presented in Table 1. 

Genotypes (varieties and lines) were phenotyped in the quality collection nursery at the Omsk SAU (55°02′ N, 73°32′ E; Omsk, Russia) in 2021. Field trials utilized a randomized complete block design with two replicates. Sowing was carried out with a SSFK-7 seeder with a sowing rate of 500 seeds per 1 m^2^ in the middle of May. The predecessor was fallow. The harvest was in late August.

The average daily temperature in May 2021 was much higher than the long-term average, while the total amount of precipitation was less than the long-term normal. In July 2021, there was less precipitation. The chemicals used in the study were of analytical grade unless stated otherwise.

The wheat genotypes were milled according to AACCI Method 26-70.01 [16] by using a Chopin CD 1 Laboratory Mill (Chopin Technologies, Villeneuve La Garenne, France). Bran samples were ground up using a Perten 3100 Laboratory Mill with a mesh of 500 μm. To obtain whole wheat flour, each sample’s bran (ground) and flour were homogeneously blended. Whole wheat flours were rested for two weeks before usage. 

### 2.2. Methods

#### 2.2.1. Determination of Grain Characteristics

The moisture contents of the grains were analyzed using the AACCI Method 44-15A [16]. Protein content analysis was performed using NIR-spectroscopy according to ISO Standard ISO 12099:2017(E) [17]. The content of wet gluten was determined according to GOST 27839-2013 [18]. Grinding of the grain was carried out on a Perten laboratory mill 3100 (Perten Instruments AB, Huddinge, Sweden) equipped with a 0.9 mm sieve. The hectoliter weight was determined using an apparatus with a one-liter container using cleaned grain and reported on an as-is moisture basis. For each sample, two determinations were made and the average was calculated with an accuracy of 1 g. The thousand kernel weight was determined as two parallels, 500 grains were counted and weighed with an accuracy of 0.01 g. 

#### 2.2.2. Determination of Farinograph Characteristics

The moisture contents of whole wheat flour samples were determined using the AACCI Method 44-15A [16]. The farinograph properties of the whole wheat flour samples were tested using a farinograph (Brabender Farinograph-AT, Duisburg, Germany) equipped with a 50-g bowl according to AACCI Method 54-21 [16]. The farinograph curve yields the development time (min.), water absorption (14% moisture base), stability (min.), softening degree (BU, 12 min. after max.), and farinograph quality number.

#### 2.2.3. Whole Wheat Bread Production and Quality Evaluation

Whole wheat bread samples were produced by using the modified AACCI Method 10-10B [16] with some modifications described in Cetiner et al. [19]. Whole wheat flour (100 g, 14% mb), yeast suspension (25 mL, 8.0%), salt solution (25 mL, 6.0%, non-iodized), and water (according to the farinograph water absorption value) were used in the formula. Doughs were mixed by using a National Mfg. pin mixer (Lincoln, NE, USA). Doughs were left for fermentation (30 min) and then punched, rounded, and molded. Fermentation temperature and humidity conditions were 30 ± 1 °C and 85% relative humidity in the fermentation cabinet. After the second fermentation (30 min), the dough was sheeted, molded, and panned. The final proof was 45 min. Preliminary studies on whole wheat breads indicated that longer proof times caused dough collapse and crust deformation, resulting in lower loaf volume. Hence, the final proof was decreased to 45 min to obtain whole wheat breads with better quality. The whole wheat breads were baked in the rotary oven (Despatch, Minneapolis, MN, USA) at 230 °C for 25 min. The baking tests were performed in duplicate and mean values were reported. 

After cooling the loaves at room temperature for 2 h, the bread volume was determined using a loaf volumeter (National Mfg, Lincoln, NE, USA) by the rapeseed displacement method according to AACCI Method 10.05-01 [16]. Whole wheat breads (duplicate samples) were placed in plastic bags and maintained at room temperature for 24 h to determine firmness. AACCI Method 74-09 [16] was used to determine the firmness of whole wheat breads. A texture analyzer (Stable Microsysytems, TA-XT plus, Godalming, Surrey, England) equipped with 50 kg load cell and a 36 mm cylinder probe was used for texture analysis. The force (firmness, gram-force) required to compress 40% of two slices (1.25 cm each) was determined at 1.7 mm/sec test speed. Crumb and crust color L*, a*, b* (D65, 10º) values of whole wheat bread samples were measured using a spectrophotometer (Miniscan by HunterLab, Reston, VA, USA) according to Method E 1164 [20]. 

A panel of three bread quality experts evaluated the bread samples’ quality features (symmetry, crust color, crumb cell structure, and softness). Bread samples were handed over in duplicate at room temperature under typical white light. The symmetry and crust color of the breads were graded on a 5-point scale (1: poor to 5: very good), while the crumb cell structure and softness of the breads were graded on a 10-point scale (1: poor to 10: very good). The final score was calculated as the mean of all panelists’ scores.

#### 2.2.4. Determination of Free, Bound, Total Phenolic Contents and Antioxidant Activities (DPPH Radical Scavenging Activity and ABTS Assay)

Whole wheat bread slices (1.25 cm thick) were dried in an oven (40 °C) for 24 h. Dried bread slices were grounded and sieved using a 35-mesh sieve. Before analyzing, the grounded samples were defatted using hexane at a ratio of 1:5 (*w*/*v*). The samples were shaken at 200 rpm for 10 min with a shaker (MK200D, Yamato Scientific Co., Ltd., Tokyo, Japan) and centrifuged for 5 min at 2500× *g* (Heraeus, Multifuge X3 FR, Thermo Scientific, Dreieich, Germany). The defatting method was done three times, and the samples were dried in a fume hood for 12 h. The extraction of free and bound phenolics from whole wheat bread samples was carried out as previously described [21]. The Folin–Ciocâlteu method was adapted to detect the concentrations of free and bound phenolic compounds, and the total phenolics were calculated using their sum. Folin–Ciocâlteu reagent (2N; 500 L), Na_2_CO_3_ solution (200 g/L; 1.5 mL), and distilled water (7.9 mL) were mixed with 100 L of methanol extract and incubated for 120 min in the dark. The sample was then centrifuged for 5 min at 4000× *g*, and the absorbance at 760 nm was measured with a spectrophotometer (Shimadzu 150 UV-1800, Kyoto, Japan). Gallic acid equivalents (GAEs) were used to calculate the phenolic content. The antioxidant capacity was measured using the DPPH radical scavenging activity method, as described by Singh et al. [22]. In this method, 100 L of extract was treated with 4.9 mL of fresh 1,1-diphenyl-2-picrylhydrazil (DPPH) solution. Following a 60-min incubation at 30 °C, the absorbance of the solution was measured at 515 nm using a Shimadzu 150 UV-1800 spectrophotometer (Kyoto, Japan). The results are given in milligrams of TE per 100 g of bread. The ABTS radical-cation scavenging capacity of the whole wheat bread extracts was determined using the method presented by Rice-Evans and Miller [23], with some modifications. To begin, 2 mL of ABTS solution was added to 100 L of extract and incubated for 6 min at 30 °C. After incubation, the solution’s absorbance at 734 nm was measured with a spectrophotometer (Shimadzu 150 UV-1800, Kyoto, Japan). The results are given in milligrams of TE per 100 g of bread.

#### 2.2.5. Total Dietary Fiber (TDF) Analysis

TDF contents of the samples were determined according to AOAC 991.43 [24]. Sequential enzymatic digestion was applied to the samples using heat-stable α-amylase, protease, and amyloglucosidase to remove digestible starch and protein. Enzyme digestate was treated with alcohol before filtering, and total dietary fiber residue was washed with alcohol and acetone, dried, weighed, and expressed as TDF%.

#### 2.2.6. Estimation of the In Vitro Glycemic Index Value of Bread Samples

The rate of in vitro starch hydrolysis of bread samples was determined using the method described by Kahraman et al. [25]. The samples were ground to pass 212 µm prior to the analysis. Bread samples were hydrolyzed with digestive enzymes (pepsin (Sigma, P7000, St. Louis, MO, USA), pancreatin (Sigma, P7545, St. Louis, MO, USA), and amyloglucosidase (3300 U/mL, Megazyme Int., Wicklow, Ireland) at 37 °C. Aliquots (100 μL) were taken into Eppendorf tubes at 0- and 90-min intervals and mixed with 1 mL of absolute ethanol. These solutions were centrifuged at 800× *g* for 10 min and glucose content was measured with glucose oxidase-peroxidase (GOPOD) reagent (Wicklow, Ireland Megazyme Int.) using a spectrophotometer (Shimadzu 1601, Tokyo, Japan) at 510 nm wavelength. The hydrolysis index (HI) shows the starch digestion rate and the estimated glycemic index (GI) indicates the digestibility of the sample against white bread as the reference sample. White bread is assumed to be digested totally and the hydrolysis index (HI) of the sample is calculated as the ratio of the hydrolyzed (digested) starch content of the sample to that of the white bread. 

The in-vitro GI was determined by using the following equation of Goñi et al. [26];

GI = 39.71 + 0.549HI

#### 2.2.7. Statistical Analysis

All experiments were performed in duplicates and the mean values were recorded. Results were analyzed using a one-way analysis of variance (ANOVA) and statistical analysis was performed with the software JMP (Version 11.0.0, SAS Institute Inc., Campus Drive Cary, NC, USA, 2013). When significant (*p* < 0.05) differences were found, the least significant difference (LSD) and *t*-test were used to determine the differences among means.

## 3. Results

### 3.1. Determination of Grain Characteristics

The characteristics of the wheat cultivars and lines used in the study are given in Table 2. The grain protein and wet gluten contents and hectoliter weights of the wheat samples were in the range of 16.7–17.7%, 34.1–38.5%, and 72.2–73.4 kg/hL, respectively. There were no significant differences among the genotypes for these parameters. The thousand kernel weights of the samples were in the range of 31.4–33.3 g and the thousand kernel weights of Element 22 and Blue 10 were significantly lower than the other samples.

### 3.2. Determination of Farinograph Characteristics

The farinograph properties (dough development time, water absorption, stability, softening degree, and quality number) of the whole wheat flour samples are given in Table 3. The high stability and water absorption with the low softening degree values of whole wheat flour are the main indicators of good bread-making quality [27]. Purple 8 and Blue 10 had relatively higher stability (3.16 and 2.86 min), lower softening degree (115 and 127 BU), and higher quality numbers (60 and 62), which are indications of their better rheological properties. The farinograph water absorption value is mainly affected by the characteristics of gluten and starch. Water absorption must be evaluated in conjunction with the other farinograph characteristics in order to be appropriately interpreted. Higher water absorption combined with a lower degree of softening suggests good quality flour, whereas lower water absorption combined with a higher degree of softening indicates poor quality. Dough development time is affected by the quality of the gluten and the degree of starch damage. The stability and the degree of softening are the gluten quality parameters which impact the viscoelastic properties of the gluten complex. In practice, higher stability and lower degree of softening indicate that the dough will be more able to sustain long mechanical processing treatments [27]. Stronger flours with higher protein content have a longer development time than weaker ones. Stability is an indication of how well a flour resists overmixing. Stronger flours are usually more stable than weaker ones from the same wheat class. The higher quality number indicates a stronger flour. In the present study, the samples had different farinograph properties because of their differences in protein content and quality. 

Blue 10 had the highest dough development time among other colored wheats. The result is in line with Šebestíková et al. [28], who found that the blue colored variety AF Oxana had the highest dough development time among fine flour samples. Burešová et al. [29] concluded that purple and blue colored wheat flour had higher stability values as compared to commercial wheat; however, no clear differences were found in the behavior of doughs prepared from colored wheat and commercial flours.

### 3.3. Whole Wheat Bread Quality Evaluation

**Figure 2:**
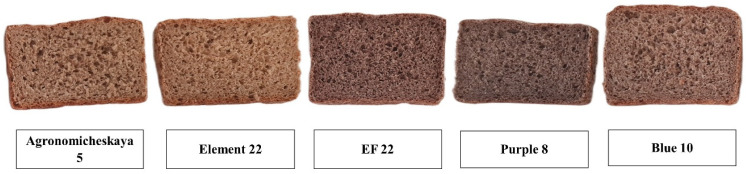
Whole wheat bread samples. (The sizes of the bread photographs should not be used to compare their volumes).

Loaf volume is one of the most important factors in determining the quality of whole wheat bread. Loaf volume values of the whole wheat breads are presented in Table 4 and the breads are shown in Figure 2. The loaf volumes of the whole wheat breads were in the range of 329–387 mL. It is known that bran fractions, if included in the flour, reduce the volume of the bread because interactions of bran particles with the gluten network cause weakening of the gluten matrix [30]. Therefore, the loaf volumes of the whole wheat bread samples were relatively low as compared to the breads produced by using white flour. The usage of whole wheat flour is limited due to poor processing and end-product quality. The presence of fiber in the dough system had a positive effect on water absorption and gassing power and a negative influence on other dough and baking quality characteristics, including farinograph stability and bread loaf volume [31]. Dilution of gluten by fiber could only account for part of the changes in mixing/baking properties of the wheat flour/fiber blends and whole wheat flours. In addition to this, the redistribution of water caused by the hydrophilic properties of dietary fibers also affects mixing/baking properties of flours. Hence, more than one mechanism seems to operate spontaneously. The contribution of different factors may vary due to the complexity of the system’s composition and the variety of structures of gluten proteins and fiber molecules. Moreover, the lean formula (no added shortening, sugar, and bread improvers) used in the breadmaking might be another reason for the relatively lower loaf volumes received in the present study. Hřivna et al. [32] evaluated breads produced using colored wheat bran and reported that volumes of 30% bran-supplemented bread samples were in the range of 264–342 mL. In the present study, the whole wheat bread produced with blue-colored wheat had the highest (*p* < 0.05) loaf volume among all whole wheat bread samples. By considering the farinograph parameters, Blue 10 had the highest dough development time, good stability value, and the highest quality number, which indicates the better bread making quality of this variety. The lowest loaf volume was determined for the whole wheat bread produced from the cv. Agronomicheskaya 5.

The quality evaluation characteristics of the whole wheat bread samples (determined by expert panelists) are presented in Table 4. Symmetry, crust color, crumb cell structure, and softness values were evaluated. Whole wheat bread produced using Blue 10 had significantly better (*p* < 0.05) symmetry, crust color, crumb cell structure, and softness values than the other whole wheat bread samples.

Texture analysis was carried out to determine the crumb firmness values of the whole wheat bread samples and the results are presented in Table 4. There were generally significant differences between the whole wheat breads for firmness values (*p* < 0.05). Whole wheat bread produced with Blue 10 had the lowest while Element 22 had the highest firmness values as compared to the other whole wheat bread samples. The lowest crumb firmness value of the bread from Blue 10 is probably due to its highest loaf volume value. The samples with lower volumes (Agronomicheskaya 5, Element 22) also had higher firmness values. Lower crumb firmness values of the breads with higher loaf volume (and vice versa) are an expected outcome and were also reported earlier by Frakolaki et al. [33]. Aoki et al. [34] also concluded that there was a significant negative correlation between the specific loaf volume and bread firmness (*p* < 0.01). As the loaf volume increases, bread crumb cells generally increase both in terms of number and size. Therefore, as the bread is compressed with the probe during texture analysis of bread crumb, the crumb, with its open network due to larger number/size of gas cells, exerts lower resistance to the probe, resulting in a lower firmness value.

Color is a key component, along with the texture and flavor of bread because of its effects on customer acceptance. Crumb and crust color values (L*, a*, and b*) of the whole wheat breads are given in Table 5. There were generally significant differences between the whole wheat breads for these traits (*p* < 0.05). The whole wheat bread produced using Element 22 had the highest L* crust and crumb color values, while Purple 8 and Ef 22 had the lowest L* crust and crumb color values as compared to the other whole wheat bread samples. Overall, the purple-grained cultivars (Purple 8 and EF 22) have a tendency to give whole wheat bread with a darker crust and crumb color. Nagyová et al. [35] evaluated consumers’ perception of bread quality in Slovakia and concluded that the consumers preferred to buy bread of the overall shape (45%) and dark crust color (25%). They also stated that consumers showed less interest in lighter crust color.

### 3.4. Free and Bound Phenolic Contents and Antioxidant Activities of the Whole Wheat Breads

Free and bound phenolic contents and antioxidant activities of the whole wheat bread samples produced using Agronomicheskaya 5, Element 22, EF 22, Purple 8, and Blue 10 are given in Table 6. The phenolic contents (free, bound, and total) of the whole wheat bread samples ranged from 348.8 to 375.5, 360.3 to 471.0, and 709.1 to 836.7 mg GAE/100 g bread, respectively. There were significant differences in the free, bound, and total phenolic content values of the whole wheat breads produced from the wheat genotypes used (*p* < 0.05). Purple 8 had the highest total phenolic content (836.7 mg GAE/100 g bread) while Agronomicheskaya 5 had the lowest value (709.1 mg GAE/100 g bread) among all samples. Yu and Beta [36] examined the impact of phenolic profiles and antioxidant properties of purple wheat varieties (cvs. Öelands hvede, Indigo, and Konini) on bread-making and the total phenolic contents were determined between 331.47 and 344.01 mg ferulic acid equivalent (FAE)/100 g bread. DPPH and ABTS methods were used to determine the antioxidant capacity of the whole wheat bread samples. 

The free, bound, and total DPPH values of whole wheat bread samples prepared using whole wheat flours of Agronomicheskaya 5, EF22, Blue 10, Purple 8, and Element 22 ranged from 18.2 to 84.4, 134.8 to 204.8, and 153.0 to 279.6 mg TE/100 g bread, respectively, whereas the ABTS values of the whole wheat bread samples were between 29.8 and 129.0 mg TE/100 g bread, 140.0 and 356.0 mg TE/100 g bread, and 169.8 and 485.0 mg TE/100 g bread, respectively (Table 6). Similar to phenolic content values, antioxidant capacity was the lowest for the whole wheat bread sample produced from Agronomicheskaya 5. 

The highest total phenolic content, DPPH, and ABTS antioxidant capacity values were obtained from the whole wheat bread sample produced from Purple 8. The highest bound DPPH antioxidant capacity value was obtained from the whole wheat bread sample produced from Blue 10. The total DPPH and ABTS antioxidant activities of Blue 10 were also quite high and ranked second after those of Purple 8. As indicated earlier, the whole wheat bread produced from blue-colored wheat had the highest (*p* < 0.05) loaf volume and better crumb and crust characteristics among all whole wheat bread samples in the present study. In terms of consumer acceptance, it is an advantage to have the best bread quality characteristics and the highest phenolic content and antioxidant capacity values in the same wheat cultivar. The colored wheats used in the present study, especially Blue 10 and Purple 8, have higher phenolic contents and antioxidant activity as compared to the white colored variety Agronomicheskaya 5. These two samples also had better bread characteristics as compared to those of the white wheat. Hence, it will be more advantageous to use such wheat samples in whole wheat bread production. 

### 3.5. Total Dietary Fiber (TDF) Content of the Samples

The TDF contents of the whole wheat flour and their respective bread samples are shown in Table 7. The TDF contents of the flour samples of Agronomicheskaya 5, EF22, Blue 10, Purple 8, and Element 22 were 16.9%, 17.2%, 17.8%, 18.8%, and 19.3%, respectively. There were generally significant differences between the total dietary fiber content of whole wheat breads (*p* < 0.05). Giordano et al. [7] stated that the wholegrain flours of pigmented wheat varieties had similar total dietary fiber levels to the ones of conventional wheat varieties. Relatively higher TDF contents of the samples might be due to their lower hectoliter weights, which were in the range of 72.2–73.4 kg/hL. The results of Ciudad-Mulero [37] are in line with the present study. They reported that the total dietary fiber contents of the whole wheat flour samples produced by using bread wheat varieties ranged between 18.2 and 19.8%.

The TDF contents of the corresponding whole wheat bread samples were 18.7%, 19.5%, 18.7%, 19.7%, and 19.6%, respectively. The differences between whole wheat bread samples in terms of total dietary fiber were not statistically significant (*p* > 0.05). TDF contents of the whole wheat bread samples were higher than the TDF contents of the corresponding whole wheat flour. This might be due to resistant starch (Type 3) formation (gelatinization and retrogradation) during bread production and cooling [38]. 

### 3.6. In Vitro Glycemic Index Values of Bread Samples

The in vitro glycemic index (GI) values of the samples are presented in Table 7. The glycemic index values of the whole wheat bread samples ranged from 86.7 to 89.5. The results showed that the differences between whole wheat bread samples in terms of glycemic index were not statistically significant. Foods are classified as low (GI ≤ 55), medium (GI 56–69), and high (GI ≥ 70) glycemic index foods [39]. All of the whole wheat bread samples can be categorized as high glycemic index food. However, their GI values are considerably lower as compared to the bread produced from white wheat flour (GI:100). The results of the present study are in agreement with Atkinson et al. [40], who systematically tabulated the published and unpublished sources of reliable GI values derived from multiple studies by different laboratories. They stated that the glycemic index values of the whole wheat breads were in the high GI group (74 ± 2). However, Ficco et al. [41] determined the estimated glycemic index of the breads obtained with the purple durum wheat and blue soft wheats to be in the range of 54–70. Different from the present study, Ficco et al. [41] concluded that the comparing the experimental breads, the relative glycemic impact of the bread was reduced by the addition of the pigmented fractions, to different extents.

## 4. Conclusions

Wheat is a major crop, commodity, and staple food all across the world. As grains are milled to flour, many of the healthy components concentrated in the bran and germ are lost. Colored wheat provides novel options for healthy wheat-based products since it offers higher anthocyanins, phenolic compounds, carotenoids, flavonoids, and antioxidant contents. In the present study, the quality of whole wheat bread samples produced using white, red, purple, and blue colored wheats were compared in terms of their quality and dietary fiber, phenolic, and antioxidant contents, as well as in vitro glycemic index. The whole wheat bread sample prepared using blue wheat flour had the highest loaf volume and the best symmetry, crust color, crumb cell structure, and softness values, among the others. The highest total phenolic content, DPPH, and ABTS antioxidant capacity values were obtained from the whole wheat bread sample produced from Purple 8. The highest bound DPPH antioxidant capacity value was obtained from the whole wheat bread sample produced from Blue 10. The total antioxidant activities (both DPPH and ABTS) of Blue 10 were also relatively high and ranked second after those of Purple 8. In terms of consumer acceptance, it is an advantage to have the best bread quality characteristics and high phenolic content and antioxidant capacity values in the same cultivar. Although there were no significant differences between the whole wheat bread samples for TDF contents and in vitro glycemic index values, their GI values were considerably lower as compared to that of white wheat bread. It can be concluded that these unconventional colored wheat varieties might be important sources of biologically active phytochemicals, and they could be valuable raw materials for the production of functional cereal products.

## Figures and Tables

**Table 1:** Wheat cultivars and lines used in the study.

Abbreviation	Cultivar/ Line	Origin	Grain Color
Agron 5	*cv* Agronomicheskaya 5	Sonata *2// TAM 200/Turaco	White
Element 22	*cv* Element 22	Granıt/ Saratovskaya 29/3/Erythrospermum 59/Tselınnaya 20//Tertsıya	Red
EF 22	*cv* EF 22	Element 22 *2/i:S29^PF^	Purple
Purple 8	line Element 22-Purple^PF^ (8)	Element 22 *2/i:S29^PF^	Purple
Blue 10	line Element_22-Blue^4Th^ (10)	s:S29_4Th/4D/Element 22	Blue

**Table 2:** Grain characteristics of wheat cultivars and lines.

Sample	Moisture Content (%)	Protein (%, grain)	Wet Gluten (%, Flour)	Thousand Kernel Weight (g)	Hectoliter Weight (kg/hL)
Agron 5	13.2	17.4 ± 1.13	36.5 ± 0.78	33.3 ± 0.85 ^a^	73.4 ± 0.57
Element 22	12.8	17.7 ± 0.35	38.5 ± 0.71	31.4 ± 1.27 ^b^	72.2 ± 1.13
EF 22	12.2	17.4 ± 0.64	34.7 ± 1.41	33.3 ± 0.85 ^a^	73.2 ± 0.57
Purple 8	11.8	16.7 ± 0.57	34.6 ± 1.13	32.9 ± 1.27 ^a^	73.0 ± 0.85
Blue 10	12.4	16.7 ± 0.78	34.1 ± 1.63	29.2 ± 0.78 ^b^	72.8 ± 1.13

Values followed by different letters in the same column are significantly different (*p* < 0.05) (if there were no significant differences between the average values, no letters were added to indicate the differences).

**Table 3:** Farinograph results of whole wheat flours.

Sample	Grain Color	Dough Development Time (Min.)	Water Absorption (14% Moisture Basis)	Stability (Min.)	Softening Degree (BU, 12 Min. after Max.)	Quality Number
Agron 5	White	2.79 ± 0.226 ^cd^	68.6 ± 0.57 ^a^	1.89 ± 0.099 ^d^	192 ± 9.9 ^a^	41 ± 4.2 ^d^
Element 22	Red	3.00 ± 0.113 ^bc^	68.0 ± 0.42 ^ab^	2.59 ± 0.071 ^c^	127 ± 7.1 ^b^	52 ± 4.2 ^bc^
EF 22	Purple	2.65 ± 0.071 ^d^	68.2 ± 0.42 ^ab^	2.61 ± 0.099 ^c^	111 ± 4.2 ^c^	50 ± 2.8 ^c^
Purple 8	Purple	3.17 ± 0.085 ^b^	67.5 ± 0.28 ^b^	3.16 ± 0.042 ^a^	115 ± 1.4 ^bc^	60 ± 1.4 ^ab^
Blue 10	Blue	4.33 ± 0.057 ^a^	66.2 ± 0.14 ^c^	2.86 ± 0.057 ^b^	127 ± 4.2 ^b^	62 ± 2.8 ^a^

Values followed by different letters in the same column are significantly different (*p* < 0.05).

**Table 4:** Volumes, firmness, and quality evaluation values of the whole wheat bread samples.

Samples	Bread Volume (mL)	Firmness (g)	Quality Evaluation			
			Symmetry	Crust Color	Crumb Cell Structure	Softness
Agron 5	329 ± 3.5 ^c^	3294 ± 37.2 ^ab^	1.4 ± 0.32 ^c^	3.3 ± 0.25 ^d^	7.2 ± 0.25 ^c^	7.4 ± 0.36 ^b^
Element 22	332 ± 7.1 ^bc^	3705 ± 184.6 ^a^	1.3 ± 0.29 ^c^	3.4 ± 0.21 ^cd^	8.4 ± 0.32 ^b^	6.3 ± 0.26 ^c^
EF 22	351 ± 14.1 ^bc^	2770 ± 304.1 ^ab^	2.3 ± 0.25 ^b^	4.3 ± 0.25 ^b^	8.3 ± 0.29 ^b^	7.3 ± 0.29 ^b^
Purple 8	355 ± 3.5 ^b^	2466 ± 173.1 ^b^	2.3 ± 0.26 ^b^	3.8 ± 0.29 ^bc^	8.3 ± 0.26 ^b^	5.2 ± 0.25 ^d^
Blue 10	387 ± 3.5 ^a^	901 ± 65.3 ^c^	3.6 ± 0.51 ^a^	5.0 ± 0.00 ^a^	9.4 ± 0.36 ^a^	8.4 ± 0.32 ^a^

Values followed by different letters in the same column are significantly different (*p* < 0.05).

**Table 5:** Crust and crumb color values of the whole wheat breads.

Samples	Crust Color of the Whole Wheat Bread			Crumb Color of the Whole Wheat Bread		
	L*	a*	b*	L*	a*	b*
Agron 5	42.06 ± 0.659 ^c^	13.99 ± 0.295 ^a^	25.64 ± 0.291 ^b^	47.28 ± 0.508 ^a^	7.65 ± 0.070 ^b^	24.67 ± 0.157 ^a^
Element 22	44.77 ± 0.313 ^a^	13.43 ± 0.096 ^b^	26.83 ± 0.044 ^a^	46.34 ± 0.261 ^a^	8.60 ± 0.102 ^a^	22.87 ± 0.261 ^b^
EF 22	40.89 ± 0.572 ^d^	11.29 ± 0.036 ^c^	21.00 ± 0.419 ^d^	36.34 ± 1.473 ^c^	7.84 ± 0.125 ^b^	15.94 ± 0.387 ^d^
Purple 8	40.38 ± 0.893 ^d^	10.79 ± 0.260 ^d^	20.93 ± 0.177 ^d^	36.80 ± 0.465 ^c^	7.31 ± 0.173 ^c^	15.34 ± 0.343 ^e^
Blue 10	43.27 ± 0.508 ^b^	11.54 ± 0.230 ^c^	23.84 ± 0.238 ^c^	43.28 ± 0.534 ^b^	7.61 ± 0.151 ^b^	18.89 ± 0.140 ^c^

Values followed by different letters in the same column are significantly different (*p* < 0.05).

**Table 6:** Phenolic contents (free, bound, and total) and antioxidant activities of the whole wheat breads.

Sample	Phenolic Content (mg GAE/100 g Bread)			DPPH (mg TE/100 g Bread)			ABTS (mg TE/100 g Bread)		
	Free	Bound	Total	Free	Bound	Total	Free	Bound	Total
Agron 5	349 ± 6.8 ^b^	360 ± 8.4 ^d^	709 ± 0.6 ^d^	18.2 ± 1.41 ^c^	134.8 ± 5.30 ^c^	153.0 ± 5.30 ^e^	29.8 ± 7.95 ^d^	140.0 ± 3.96 ^e^	169.8 ± 11.03 ^e^
Element 22	369 ± 0.0 ^a^	398 ± 1.8 ^b^	766 ± 3.6 ^b^	23.7 ± 1.77 ^c^	139.1 ± 2.47 ^c^	162.7 ± 4.94 ^d^	78.9 ± 2.38 ^c^	168.0 ± 6.79 ^c^	246.9 ± 9.14 ^d^
EF 22	376 ± 4.8 ^a^	383 ± 1.2 ^c^	758 ± 1.8 ^c^	79.9 ± 2.82 ^a^	153.8 ± 2.12 ^b^	233.7 ± 0.70 ^c^	117.3 ± 5.56 ^ab^	272.0 ± 14.71 ^c^	389.3 ± 9.17 ^c^
Purple 8	366 ± 4.2 ^a^	471 ± 1.8 ^a^	837 ± 2.4 ^a^	84.4 ± 3.17 ^a^	195.3 ± 3.17 ^a^	279.6 ± 0.00 ^a^	129.0 ± 5.09 ^a^	356.0 ± 1.13 ^a^	485.0 ± 3.96 ^a^
Blue 10	370 ± 0.6 ^a^	401 ± 0.0 ^b^	771 ± 1.6 ^b^	57.1 ± 1.41 ^b^	204.8 ± 6.71 ^a^	261.9 ± 3.88 ^b^	104.9 ± 2.55 ^b^	305.2 ± 13.58 ^b^	410.1 ± 3.99 ^b^

Values followed by different letters in the same column are significantly different (*p* < 0.05).

**Table 7:** In vitro glycemic index (GI) value of whole wheat bread samples and TDF contents of the flour and whole wheat bread samples.

Sample	TDF_Whole Wheat Flour_ (%)	TDF_Whole Wheat Bread_ (%)	GI*_Whole Wheat Bread_
Agron 5	16.9 ± 0.25 ^c^	18.7 ± 0.32	87.5 ± 0.69
Element 22	19.3 ± 0.44 ^a^	19.6 ± 0.88	86.7 ± 0.68
EF 22	17.2 ± 0.58 ^c^	19.5 ± 0.40	89.5 ± 1.82
Purple 8	18.8 ± 0.41 ^ab^	19.7 ± 0.50	88.1 ± 0.00
Blue 10	17.8 ± 0.51 ^bc^	18.7 ± 0.64	87.1 ± 1.08

GI of control bread samples produced from white wheat is assumed as 100. Values followed by different letters in the same column are significantly different (*p* < 0.05) (if there were no significant differences between the average values, no letters were added to indicate the differences).

## Data Availability

The data presented in this study are available on request from the corresponding author.
